# Improved Sterilization of Sensitive Biomaterials with Supercritical Carbon Dioxide at Low Temperature

**DOI:** 10.1371/journal.pone.0129205

**Published:** 2015-06-12

**Authors:** Anne Bernhardt, Markus Wehrl, Birgit Paul, Thomas Hochmuth, Matthias Schumacher, Kathleen Schütz, Michael Gelinsky

**Affiliations:** 1 Centre for Translational Bone, Joint and Soft Tissue Research, Medizinische Fakultät, Technische Universität Dresden, Dresden, Germany; 2 wfk—Cleaning Technology Institute e.V., Krefeld, Germany; National University of Ireland, Galway (NUI Galway), IRELAND

## Abstract

The development of bio-resorbable implant materials is rapidly going on. Sterilization of those materials is inevitable to assure the hygienic requirements for critical medical devices according to the medical device directive (MDD, 93/42/EG). Biopolymer-containing biomaterials are often highly sensitive towards classical sterilization procedures like steam, ethylene oxide treatment or gamma irradiation. Supercritical CO_2_ (scCO_2_) treatment is a promising strategy for the terminal sterilization of sensitive biomaterials at low temperature. In combination with low amounts of additives scCO_2_ treatment effectively inactivates microorganisms including bacterial spores. We established a scCO_2_ sterilization procedure under addition of 0.25% water, 0.15% hydrogen peroxide and 0.5% acetic anhydride. The procedure was successfully tested for the inactivation of a wide panel of microorganisms including endospores of different bacterial species, vegetative cells of gram positive and negative bacteria including mycobacteria, fungi including yeast, and bacteriophages. For robust testing of the sterilization effect with regard to later application of implant materials sterilization all microorganisms were embedded in alginate/agarose cylinders that were used as Process Challenge Devices (PCD). These PCD served as surrogate models for bioresorbable 3D scaffolds. Furthermore, the impact of scCO_2_ sterilization on mechanical properties of polysaccharide-based hydrogels and collagen-based scaffolds was analyzed. The procedure was shown to be less compromising on mechanical and rheological properties compared to established low-temperature sterilization methods like gamma irradiation and ethylene oxide exposure as well as conventional steam sterilization. Cytocompatibility of alginate gels and scaffolds from mineralized collagen was compared after sterilization with ethylene oxide, gamma irradiation, steam sterilization and scCO_2_ treatment. Human mesenchymal stem cell viability and proliferation were not compromised by scCO_2_ treatment of these materials and scaffolds. We conclude that scCO_2_ sterilization under addition of water, hydrogen peroxide and acetic anhydride is a very effective, gentle, non-cytotoxic and thus a promising alternative sterilization method especially for biomaterials.

## Introduction

Sterilization of tissue graft materials is an important issue since those materials are often composed of organic natural or synthetic polymers being sensitive to high temperatures. Low temperature sterilization methods like gamma irradiation and electron beam treatment, as well as ethylene oxide gas (EO) and low-temperature formaldehyde/steam exposure have been shown to alter morphology, structure, and surface properties of different organic polymers [1–4]. An alternative method for the inactivation of microorganisms is the application of dense phase and supercritical carbon dioxide (scCO_2_) [5–7]. ScCO_2_ offers many advantages as a sterilization agent, since it is non-toxic, non-reactive, has high penetration ability and is easily removable by depressurization [8]. Furthermore, due to the non-reactive nature of CO_2_, supercritical treatment does not compromise the morphology, structure and mechanical properties of the biomaterials. Different gram positive and gram negative vegetative bacteria have been successfully inactivated by scCO_2_ treatment [6, 8] however the inactivation of bacterial endospores using scCO_2_ treatment requires either high temperatures and pressure or prolonged incubation time [9, 10]. These harsh conditions are not suitable for sensitive biomaterials like polysaccharides, tissue samples or proteins. Supplementation of the scCO_2_ process with low amounts of volatile additives has been shown to increase the inactivation rate of bacterial endospores [7]. Suitable additives which allow the effective inactivation of bacterial endospores are peroxides, carboxylic acids, alcohols and mixtures thereof. For the sterilization of medical devices a guaranteed sterility assurance level (SAL) is required. For most of the medical devices a SAL of 10^–6^ according to EN 556–1 [11] is applied. Different groups have demonstrated the potential of scCO_2_ combined with low amounts of additives to reach the required SAL for bacterial spores without high temperature and pressure (Table 1).

**Table 1:** Overview on publications describing the potential of scCO_2_ combined with low amounts of additives to reach the required SAL of 10^–6^ for bacterial spores without high temperature and pressure (TFA = trifluoracetic acid, PAA = peracetic acid).

author (year)	bacterial spores tested	effective additives (log reduction>6)	pressure, temperature, duration	additives tested, but not effective enough
White (2006) [7]	*G*. *stearothermophilus B*. *subtilis*	PAA (0.002%), H_2_O (0.15%); TFA	9.65 MPa, 35°C, 60 min	ethanol, citric acid, succinic acid, H_2_O_2_, formic acid, acetic acid, malonic acid
Zhang (2006) [12]	*B*. *pumilis*	H_2_O_2_ (0.0002%)	27.5 MPa, 60°C, 4 h	ethanol, isopropanol
Hemmer (2007) [10]	*G*. *stearothermophilus B*. *atrophaeus*	H_2_O_2_ (0.6%)	30.4 MPa, 40°C, 4 h	-
Shieh (2009) [13]	*B*. *pumilis*	H_2_O/methanol/formic acid (3.3%/0.33%/0.033%), H_2_O/methanol/formic acid/H_2_O_2_ (3.3%/0.33%/0.033%, 0.11%) (log reduction 5)	10.13 MPa, 50°C, 45 min (10 dynamic, 30 static, 5 dynamic)	H_2_O_2_, Triton X-100, tert butyl hydroperoxide, mixtures of methanol and formic acid or H_2_O_2_ without water
Checinska (2011) [14]	*B*. *pumilis*	H_2_O/H_2_O_2_ (3.3%/0.1%)	8.1 MPa, 50°C, 30 min (10 dynamic, 15 static, 5 dynamic)	-
Donati (2012) [15]	*G*. *stearothermophilus*	H_2_O_2_ (0.0002–0.0006%)	27 MPa, 40°C, 90–30 min	PAA (0.0002%)
Park (2013) [16]	*B*. *cereus*	ethanol (2%)	10 MPa, 60°C, 90 min	-

All investigations summarized in Table 1 were performed using spores on paper strips or metallic surfaces. However, the inactivation of microorganisms inside of three-dimensional biomaterials or tissue samples is of higher relevance for the effective sterilization of implants. Karajanagi and co-workers [17] inoculated polyethylene glycol hydrogels with different microorganisms including spores of *B*. *subtilis*. Since no additives were applied, an adequate microbial inactivation was achieved only after long incubation time (4 and 6 hours) and high temperature (70°C). Another study described the inoculation of different vegetative bacteria and spores of *B*. *atrophaeus* into porcine acellular dermal matrix [18]. Sterilization with scCO_2_ under addition of 0.0055% peracetic acid (PAA) reduced spore content to 10^–6^ after 27 min of treatment. Herdegen *et al*. [19] sterilized collagen fleeces inoculated with spores of *B*. *atrophaeus* with scCO_2_. Reproducible sterility was achieved at an effective H_2_O_2_ concentration above 300 mg/l and the sterilization process was intensified by pressure cycling [19].

Several studies analyzed the impact of scCO_2_ sterilization on the mechanical properties of decellularized tissues, especially bone and tendon. Nichols et al. [20] studied the biomechanical properties of allografts (human tendon and cortical bone) and observed better biomechanical properties after scCO_2_ sterilization compared to gamma irradiation. Similar results were obtained by Russell and co-workers when analyzing the mechanical properties of gamma irradiated versus scCO_2_ sterilized rabbit cortical bone [21]. Furthermore, these authors demonstrated, that scCO_2_ sterilization with different additives did not compromise mechanical properties of bovine cortical bone [22] and it was demonstrated that scCO_2_ sterilized bone chips are capable of healing a critical sized tibial defect in a rabbit [23]. Baldini *et al*. reported that gamma irradiation and scCO_2_ did not influence strength of human tendon allografts however the stiffness of scCO_2_ treated samples was significantly lower compared to gamma irradiated allografts [24]. The application of scCO_2_ technology (in combination with peracetic acid as additive) for the sterilization of various allograft tissues has been successfully commercialized (NovaSterilis, NY). A basic patent on scCO_2_ sterilization of materials, particularly polymers for drug delivery and implantation was issued 1999 based on the work of Langer and co-workers [25]. Ongoing research in the field of sterilization of biomedical devices is reflected by several further patents [26]. Only few investigations were performed on the impact of scCO_2_ sterilization of synthetic bioresorbable materials. Donati and co-workers [15] analyzed the influence of scCO_2_ (with H_2_O_2_ as additive) on biomedically applied plastic materials and bioactive coatings and detected only small variations in the mechanical properties of the treated materials. However, studies on the impact of scCO_2_ sterilization of biomaterials on cellular adhesion and viability are not yet reported.

In the present study a modified scCO_2_ sterilization process under addition of H_2_O_2_ in combination with acetic anhydride has been evaluated. We investigated the inactivation of a wide panel of microorganisms including bacterial spores after embedding the microorganisms into alginate/agarose cylinders and sealing them into pouches of tyvek/foil to simulate the conditions for sterilization of bulk biomaterials in a sterile barrier package. Subsequently, the impact of scCO_2_ sterilization on the mechanical properties of hydrogels and collagen-based implant materials was analyzed. Finally, we wanted to address the question, whether scCO_2_ treatment of alginate- and collagen-based scaffolds alters the biological response *in vitro* due to possible toxic additive residuals or structural changes of the materials.

## Materials and Methods

### Sterilization

#### Process Challenge Devices (PCD)

**Fig 1:**
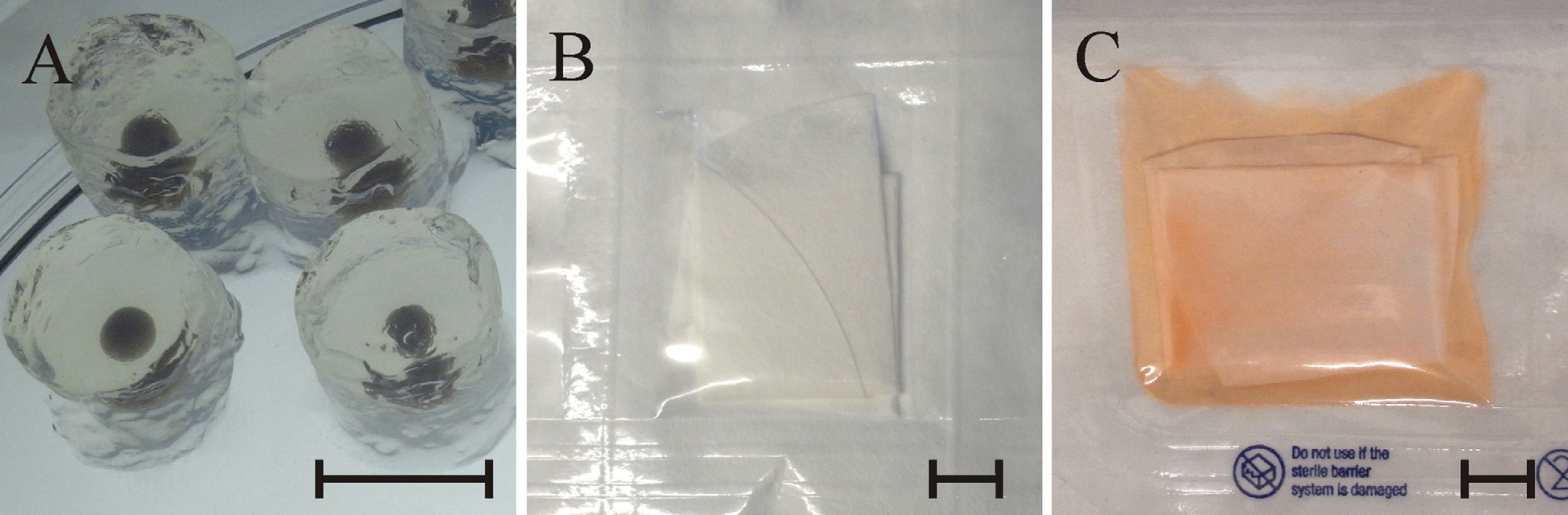
10^8^–10^9^ test microorganisms embedded in alginate/agarose cylinders (A). Sudan Red G as indicator for liquid CO_2_ (18°C, 5,5 MPa) (B) and scCO_2_. (34–38°C, ~7.3 – ~ 9,0 MPa) (C). Scale bares represent 10 mm.

For investigations on microbial inactivation the test microorganisms were embedded in alginate/agarose cylinders. These cylinders were used as Process Challenge Devices (PCD) to simulate the sterilization of 3D scaffolds. First, 10^8^–10^9^ of microorganisms were spun down and suspended in 500 μl of 2% alginate solution to form a sphere after cross-linking with 0.5 M calcium chloride solution. Spheres were further embedded in 2% agar-agar using a sandwich strategy, yielding cylinders with a diameter of 10 mm containing a central depot of microorganisms (Fig 1A). Finally the PCD were sealed into tyvek/foil pouches to implement a sterile barrier packaging system according to EN 556–1 [11]. For the scCO_2_ treatment PCD, scaffolds or material powders were sealed into tyvek/foil pouches and subjected to scCO_2_ for various incubation times.

For the quantification of surviving microbial cells the PCD were removed from the autoclave and transferred each to small plastic bags (Whirl-Pak, Nasco). After addition of 2 ml 0.9% NaCl-solution the PCD were stomached (rubbed by hand) for 20 min until a homogeneous suspension was obtained. After preparing serial dilutions in 0.9% NaCl-solution the suspensions were plated out on CASO-agar. The number of surviving cells was determined by counting colony forming units (CFU).

#### Indicator for scCO_2_

An indicator was developed to monitor the phase transition of liquid CO_2_ into the supercritical state (scCO_2_). A 20x20 mm piece of filter paper (Machery Nagel, MN 640W, 90 mm diameter) was soaked in a Sudan Red G (Sigma Aldrich) solution (1.5 mg/ml in ethanol), dried, soaked again. After drying the stained filter paper was wrapped in a 90 mm filter disc and sealed in two tyvek/foil pouches, (Fig 1B). In the case of successful transition of liquid CO_2_ into the supercritical state the dye was dissolved and became visible through the foil of the tyvek pouches (Fig 1C). Each scCO_2_ sterilization process was supplemented with such a scCO_2_-indicator.

#### ScCO_2_ treatment

ScCO_2_ treatment was performed in a 750 ml stainless steel autoclave (J&W Scientific, Inc.) equipped with inlet and outlet valve, pressure gauge and safety valve (P_max_: 10.0 MPa). The PCD, scaffold precursor powders or scaffolds were sealed into tyvek/foil pouches, inserted into the autoclave and the additive mixture was pipetted to the bottom of the autoclave. Subsequently, the autoclave was filled with CO_2_ (300 g liquid CO_2_, 18°C, 5.5 MPa). By heating the autoclave the liquid CO_2_ was transferred to the supercritical state (~38°C, ~8.5 MPa). PCD were subjected to scCO_2_ treatment for 5, 15, 30 and 45 min, scaffold precursor powders and scaffolds for 45 min at ~8.5 MPa and ~38°C. All samples were treated in the presence of 0.25% water/0.15% H_2_O_2_/0.5% acetic anhydride (Sigma). Depressurization was performed in all cases within 6,5 min.

#### Classical sterilization methods

For comparison biomaterials and precursor powders were sterilized by steam (121°C, 20 min, D23 autoclav, Systec, Germany), gamma irradiation (> 25 kGy, ^60^Co) (BBF Sterilization Service, Kernen, Germany), ethylene oxide (EO) (Central sterilization unit, University Hospital Dresden), and formaldehyde/steam (Webeco GmbH).

### Materials preparation

#### Alginate hydrogel cylinders

Sodium alginate from brown algae (Sigma), sterilized by steam, gamma irradiation or scCO_2_ treatment was dissolved in deionized water as 2% solution. For each sample the alginate solution was cross-linked in a 10 ml glass beaker after spraying with 1 M CaCl_2_ solution (Sigma) to build an initial alginate gel membrane at the surface. Subsequently, the residual CaCl_2_ solution was carefully overlaid. After incubation for 66 h at RT the gelation of the cylinders was complete, and the stable gels were removed from the beakers. Samples were cut to a final height of 16 mm.

#### Plotting

Alginate powder (Sigma) was sterilized by steam sterilization. 3% alginate solution was prepared in PBS and 9% methylcellulose (MC, Fluka), sterilized by gamma irradiation or scCO_2_ treatment was added and allowed to swell for additional 2 h at RT. After final stirring, the polysaccharide blend was plotted with the 3D Bio-Scaffold Printer (BioScaffolder 2.1, Gesim, Germany). Cross-linking of the final construct was achieved by soaking in a 100 mM CaCl_2_ solution for 30 min.

#### Collagen-based scaffolds

Mineralized collagen was fabricated like published previously [27]. Shortly, acidic bovine collagen I (Syntacoll, Germany) solution was neutralized with TRIS buffer, containing calcium and phosphate ions. Synchronous refibrillation and mineralization was executed at 37°C for about 16 hours. Mineralized collagen was collected by centrifugation and lyophilized in 96-well plates to obtain cylindrical scaffolds with 6 mm diameter. Lyophilized scaffolds were chemically cross-linked with 1 wt-% EDC in 80 vol-% ethanol and after some washing steps freeze-dried a second time. Finally, the scaffolds were cut to a height of 8 mm for analysis of mechanical properties and of 3 mm for cell culture experiments.

Biphasic collagen scaffolds (Matricart) were provided by Jotec GmbH.

Collagen-based scaffolds were subjected to sterilization in their final state.

### Mechanical properties of sterilized materials

Mechanical properties of alginate scaffolds from differently sterilized alginate powders and differently sterilized mineralized collagen scaffolds were analyzed by uniaxial compression using a Z010 (Zwick, Germany). Alginate hydrogel scaffolds (n = 8; diameter = 15 mm; height = 16 mm) were statically compressed with a velocity of 0.1 mm/sec.

The samples of mineralized collagen (n = 12; diameter = 6 mm; height = 8 mm) were incubated in simulated body fluid (SBF [28]) 24 h prior to testing and static compression was conducted with a velocity of 1%/s. Data were analyzed concerning compressive modulus at the linear slope.

**Fig 2:**
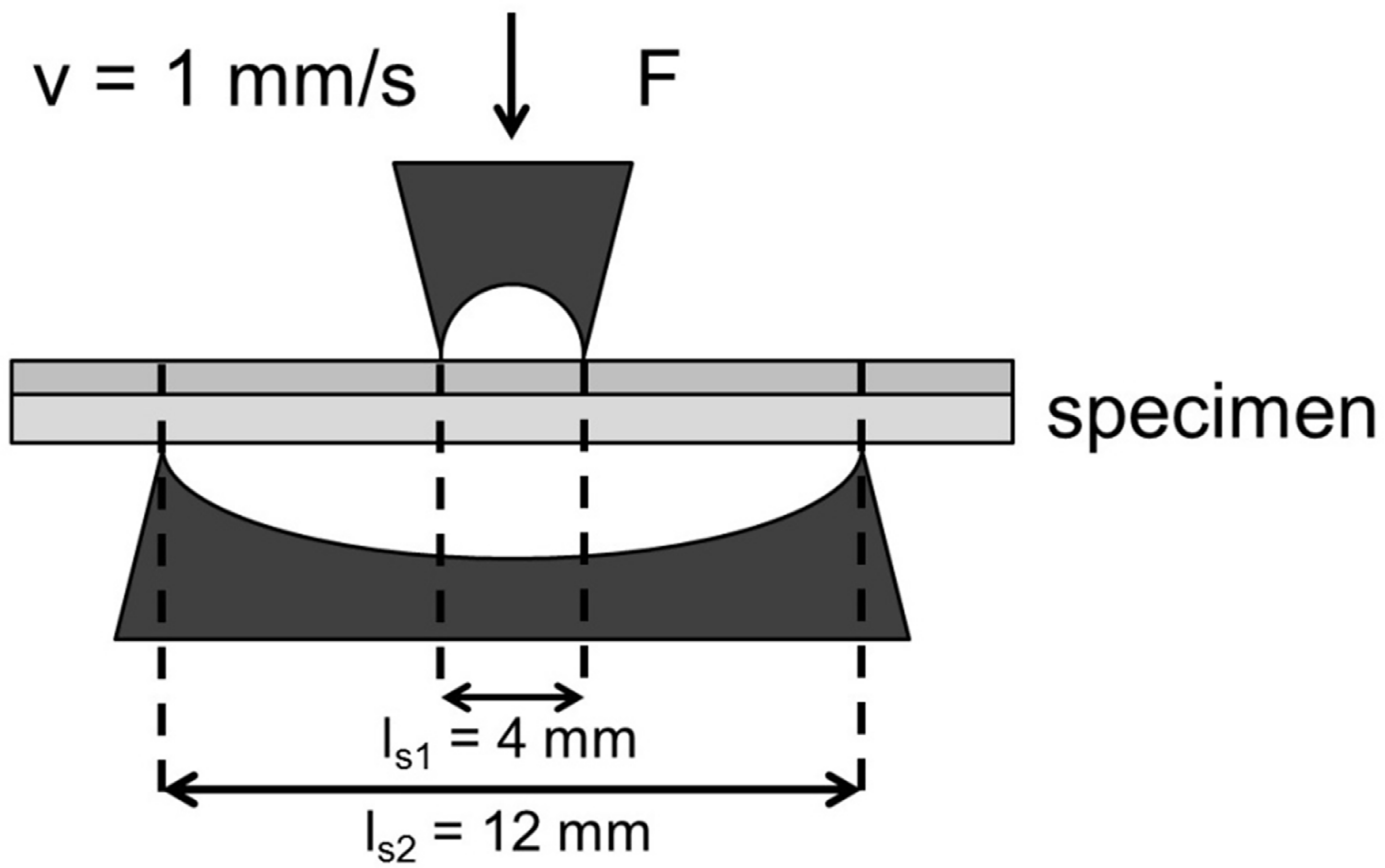
Schematic drawing of the custom-made 4-point bending apparatus.

Bending strength of untreated and differently sterilized biphasic collagen scaffolds (Matricart) was measured using the same testing machine equipped with a custom-made four-point bending apparatus (Fig 2) with 4mm and 12mm inner and outer span of the contact points, respectively, and a crosshead speed of 1mm/min. Samples (3x1.8x20 mm, n = 6) were placed in the testing device with their dense layer on top. Bending stress was calculated at 5, 10 and 20% deformation according to Eq E1 with *F*_*x*_ being the bending force at x% deformation, *l* being the distance between the inner and outer supports and *W* being the cross section coefficient.

$$\sigma_{B}=\frac{F_{x}l}{2W}\;\text{with}\;W=\frac{1}{6}bh²\;\text{and}\;l=\frac{l_{s2}-l_{s1}}{2}$$ (E1)

Rheological properties of the methylcellulose/alginate pastes (n = 2) were determined at room temperature using a Physica MCR 300 rheometer (Anton Paar, Austria) with plate-plate geometry (plate diameter 50 mm, gap 1 mm). Using a controlled shear stress mode (stress ramp from 1 to 2250 Pa) shear rate was measured and viscosity was calculated.

### Cell culture

Bone marrow derived human mesenchymal stem cells (hMSC) from the iliac crest of two healthy male donors (age: 30–37 years) were kindly provided by the group of Prof. Martin Bornhäuser (Medical Clinic I, University Hospital Dresden). Written informed consent from the donors was obtained for the use of these samples in research. All procedures were approved by the Ethical Commission of the Faculty of Medicine of Technische Universität Dresden. Expansion of the cells up to passage 5 and cultivation of cell seeded materials was performed in DMEM (Gibco) supplemented with 10% fetal calf serum (FCS, Biochrom) from a previously selected lot tested as suitable for hMSC proliferation, 2 mM L-glutamine, 100 U/ml penicillin and 100 μg/ml streptomycin (all from Biochrom).

For preparation of alginate beads with embedded hMSC 50 μl cell suspension containing 5x10^4^ cells was mixed with 450 μl 2% alginate sol in PBS, prepared from alginate powder after sterilization with steam or scCO_2_ and extruded with a sterile 23G needle into a sterile 100 mM CaCl_2_ solution to form beads. After incubation for 10 min, the beads were washed with cell culture medium and finally provided with 500 μl of fresh medium.

Seeding of scaffolds from mineralized collagen (d = 6 mm, h = 3 mm) was performed after sterilization with EO, gamma irradiation or scCO_2_. The scaffolds were seeded and cultivated as described earlier [29]. Briefly, 50 μl cell suspension containing 5x10^4^ cells was applied to the top of each scaffold after 24 h pre-incubation of the scaffolds in cell culture medium and soaking of excess medium with sterile filter paper. After 30 min incubation, 500 μl medium were added to the cell-seeded scaffolds. After 5 days of cultivation the medium was additionally supplemented with 10^–7^ M dexamethasone, 5 mM β-glycerophosphate and 12.5 μg/ml ascorbic acid-2-phosphate (all from Sigma) to induce osteogenic differentiation. The whole experiment was repeated with cells of another donor.

### Cell viability, proliferation and osteogenic differentiation

#### LDH activity and MTT staining

One day after formation of alginate beads with embedded hMSC the culture medium was supplemented with 1.2 mM 3-(4,5-dimethylthiazol-2-yl)-2,5-diphenyltetrazolium bromide (MTT) (Sigma) followed by further incubation at 37°C for 4 h. The formation of dark blue formazan dye converted from MTT by mitochondrial dehydrogenases of living cells was documented using a stereomicroscope (Leica, M 205 C). Further samples of alginate beads with embedded cells (n = 4 per group) were washed twice with PBS, frozen at -80°C and thawed. Lysis was performed using 1% Triton X-100 in PBS for 30 min at 37°C. LDH activity was determined using the Cytotox96 kit (Promega) according to manufacturer`s instructions. A calibration line was constructed from 1x10^4^, 2x10^4^, 4x10^4^ and 8x10^4^ cells which were lysed in the same way to allow calculation of cell number from the LDH activity.

#### ALP activity and DNA content

After 1, 14 and 28 days of cultivation cell-seeded scaffolds from mineralized collagen (n = 3 per group) were washed twice with PBS, and frozen at -80°C in 2 ml Nalgene tubes containing six ceramic beads (Peqlab, Germany). After thawing, 450 μl of PBS were added and the samples were homogenized (2 × 10 s at 5900 rpm, Precellys24, Peqlab). After addition of 50 μl 10% Triton X-100, the suspension was further incubated on ice for 50 min. 2x10 μl of each sample were applied for DNA analysis using the QuantiFluor dsDNA system (Promega) according to manufacturer`s instructions. Again a calibration line was constructed from 1x10^4^, 2x10^4^, 4x10^4^, 8x10^4^ and 16x10^4^ cells which were lysed for 50 min in the presence of 1% Triton X-100 on ice. ALP activity was quantified by incubating 20 μl of each sample with 80 μl substrate solution (1 mg/ml 4-nitrophenylphosphate in 0.1 M diethanolamine, 0.1% Triton X-100, 1 mM MgCl_2_, pH 9.8—all from Sigma) for 30 min at 37°C. After stopping the enzymatic reaction with 1 M NaOH, absorbance at 405 nm was determined using a microplate reader (Spectra Fluor Plus, Tecan). A calibration line was established using different concentrations of 4-nitrophenol. ALP activity was related to cell number to obtain specific ALP activity.

### Statistical analysis

Figures show mean ± standard deviation. Statistical significance was evaluated with one way ANOVA and Tukey post hoc test (Origin).

## Results

### Inactivation of microorganisms embedded in alginate hydrogel

**Fig 3:**
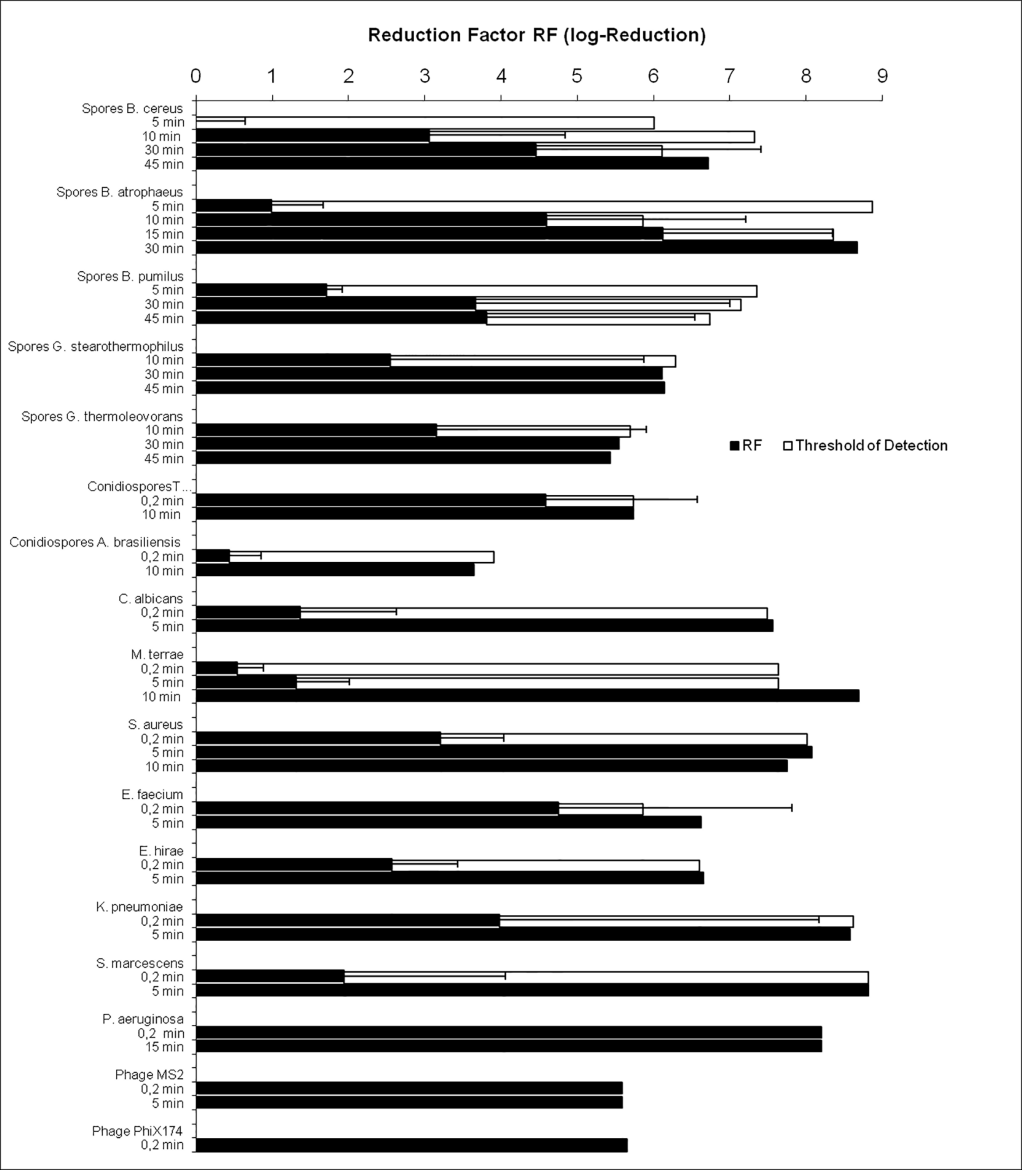
Inactivation of different microbial cells embedded into alginate/agarose PCD due to scCO_2_ treatment for different time spans. The inactivation is displayed as Reduction Factors, RF-values, giving the logarithmic reduction of surviving cells (black bars). Each experiment was performed in triplicate at minimum (n = 3–10). The threshold of detection, i.e. the maximum number of cells that could be quantified for each batch of PCD is given as white bar. For RF-values that reach the threshold of detection no standard deviation (line) is given.

The number of living cells of all test microorganisms in the alginate/agarose PCD sealed into tyvek/foil sterile barrier packaging systems was reduced by treatment in scCO_2_ under addition of 0.25% water/0.15% H_2_O_2_/0.5% acetic anhydride (Fig 3). Vegetative bacteria, fungi and bacteriophages were rapidly inactivated within several minutes. Higher process duration was required for the inactivation of bacterial endospores. Nevertheless, 4 of 5 species of bacterial endospores were inactivated with a logarithmic reduction factor of 6 or above after 30 or 45 min of scCO_2_ treatment. Only spores of *B*. *pumilis* showed lower inactivation rates in some experiments. However variances in the experiments with *B*. *pumilis* spores were quite high. According to already published results (Table 1) *B*. *pumilis* spores are difficult to inactivate and longer operating times might be required to reach a reliable inactivation. Similar inactivation results were obtained when the same panel of microorganisms was inoculated into porous scaffolds from mineralized collagen (d = 6 mm, h = 3 mm; data not shown). The following experiments on the impact of scCO_2_ on biomaterials and scaffolds were performed under the same conditions applied for the inactivation studies with PCD (8.5 MPa/38°C in the presence of 0.25% water/0.15% H2O2/0.5% acetic anhydride). The longest tested inactivation time (45 min) was chosen for all following experiments to work under conditions sufficient for the inactivation of bacterial spores.

### ScCO_2_ sterilization of polysaccharides—impact on mechanical properties of the resulting hydrogels

#### Alginate hydrogels

**Fig 4:**
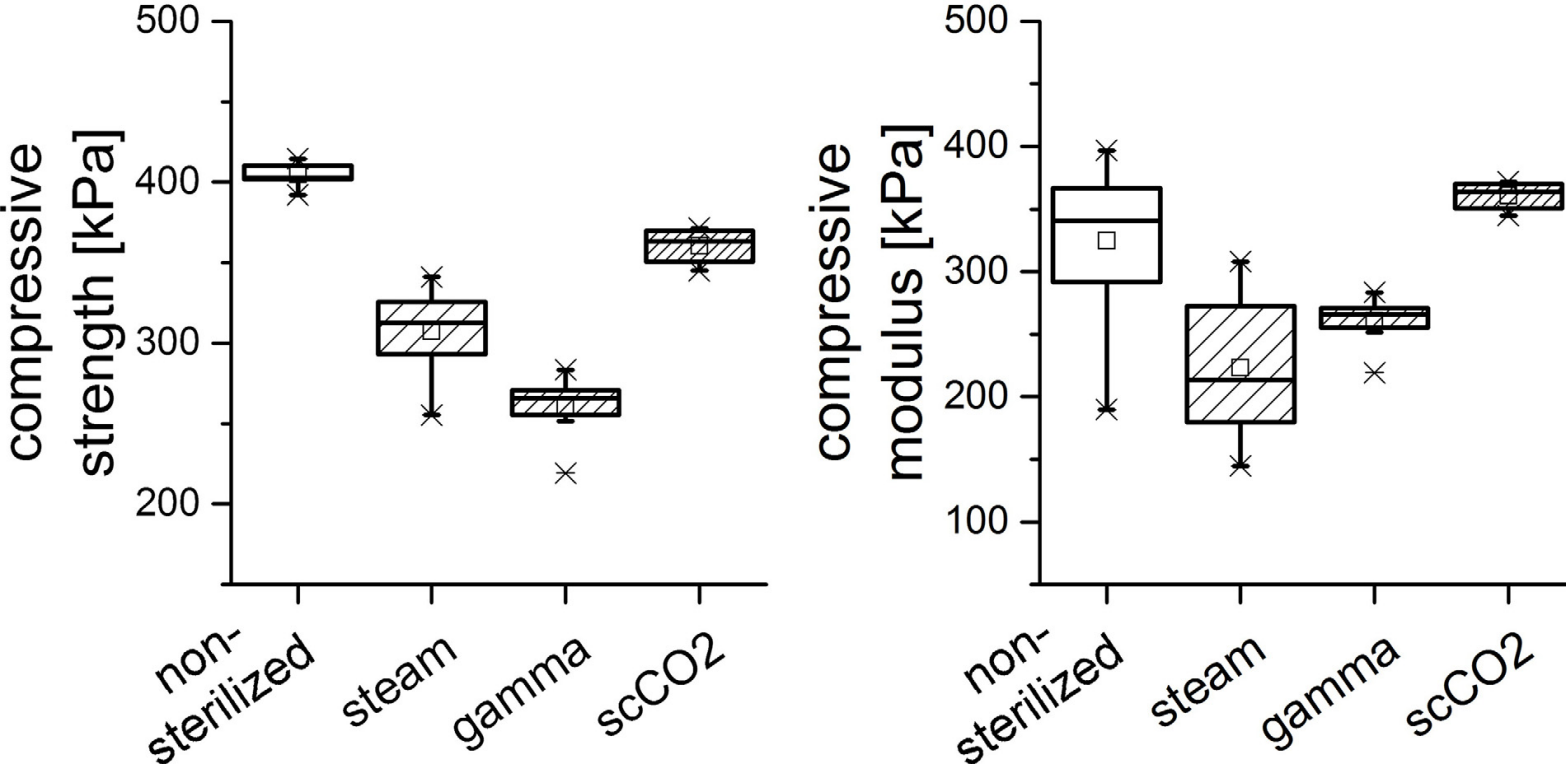
Box plots visualizing the compressive strength (a) and modulus (b) of alginate hydrogel scaffolds obtained from untreated (non-sterile) and differently sterilized alginate powders. Each box shows the 25th to 75th percentile of the measured strength and modulus, respectively (n = 6). Squares (□) represent mean values, horizontal bars inside the box show the median value, while upper and lower bars indicate the upper and lower values within 1.5 times the inter-quartile range from the upper and lower quartile (adjacent values are labelled as ×).

Hydrogel samples formed from sodium alginate, which had been sterilized as powder by steam, gamma irradiation or scCO_2_ were analyzed for compressive strength and compressive modulus. As expected, highest values for both compressive strength and compressive modulus were obtained for hydrogels from non-sterilized alginate powder. Lowest compressive strength (64.4% compared to the non-sterilized samples) was found after gamma irradiation of the alginate. Compressive strength of hydrogels from scCO_2_ treated alginate was significantly (p<0.01) higher compared to both steam and gamma sterilization and reached 89.1% compared to the non-sterilized samples. Compressive modulus of scaffolds from scCO_2_ treated alginate was not significantly changed compared to non-treated alginate samples while gamma irradiation and steam sterilization lead to decreased compressive moduli of the resulting hydrogels (Fig 4).

#### Methylcellulose/alginate plotting pastes

**Fig 5:**
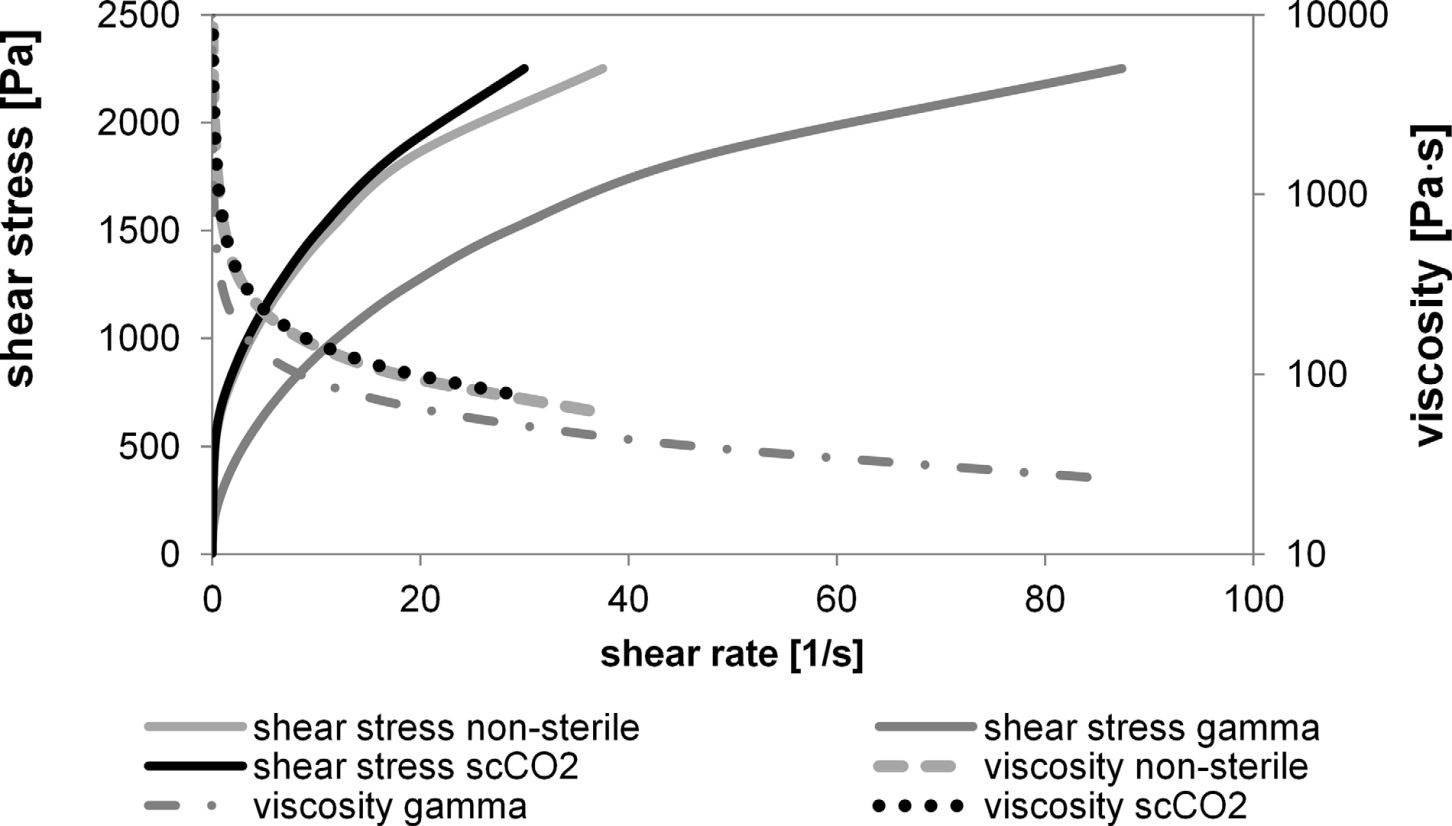
Rheological parameters of alginate/MC pastes, n = 2.

Pastes from steam sterilized alginate and differently treated (non-sterilized, gamma irradiated, scCO_2_ treated) methylcellulose (MC) (1:3) were analyzed for rheological parameters (Fig 5, Table 2). Viscosity of the samples decreased dramatically after gamma irradiation of the MC powder, while scCO_2_ treatment barely changed the rheological parameters of the pastes. Steam sterilization was not applied for methylcellulose, since it gels and agglutinates at elevated temperature.

**Table 2:** Rheological parameters of alginate/MC pastes.

shear rate	stress ramp	viscosity	
[1/s]	[Pa]	[Pa^.^s]	
0.00777	**28.9**	3.720	gamma
0.000789	**28.9**	36.600	non sterile
0.000701	**28.9**	41.200	scCO2
52.5	**1.850**	35.2	gamma
18.5	**1.850**	99.7	non-sterile
17.5	**1.850**	106	scCO2

**Fig 6:**
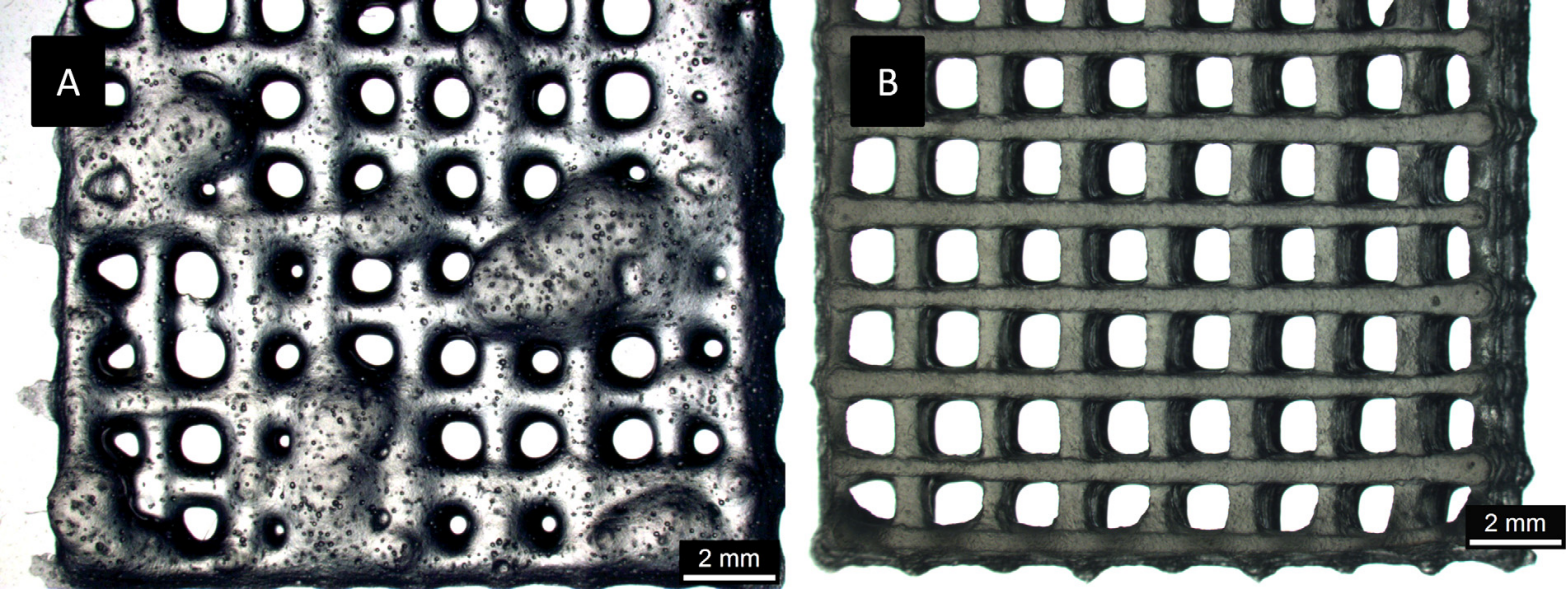
Plotted strands of methylcelulose/alginate paste (3:1), 9 x 9 strands, strand width 0.78 mm, A) gamma irradiated MC, B) scCO_2_ sterilized MC.

The examined pastes were furthermore tested for their suitability for a 3D plotting process. MC-based plotting pastes from scCO_2_ treated precursor powders showed excellent plotting behavior (Fig 6B): the gels were homogenously extruded from the printing nozzle and formed stable strands maintaining the desired scaffold shape.

### Mechanical properties of collagen-based scaffolds after scCO_2_ sterilization

#### Porous scaffolds from mineralized collagen

**Fig 7:**
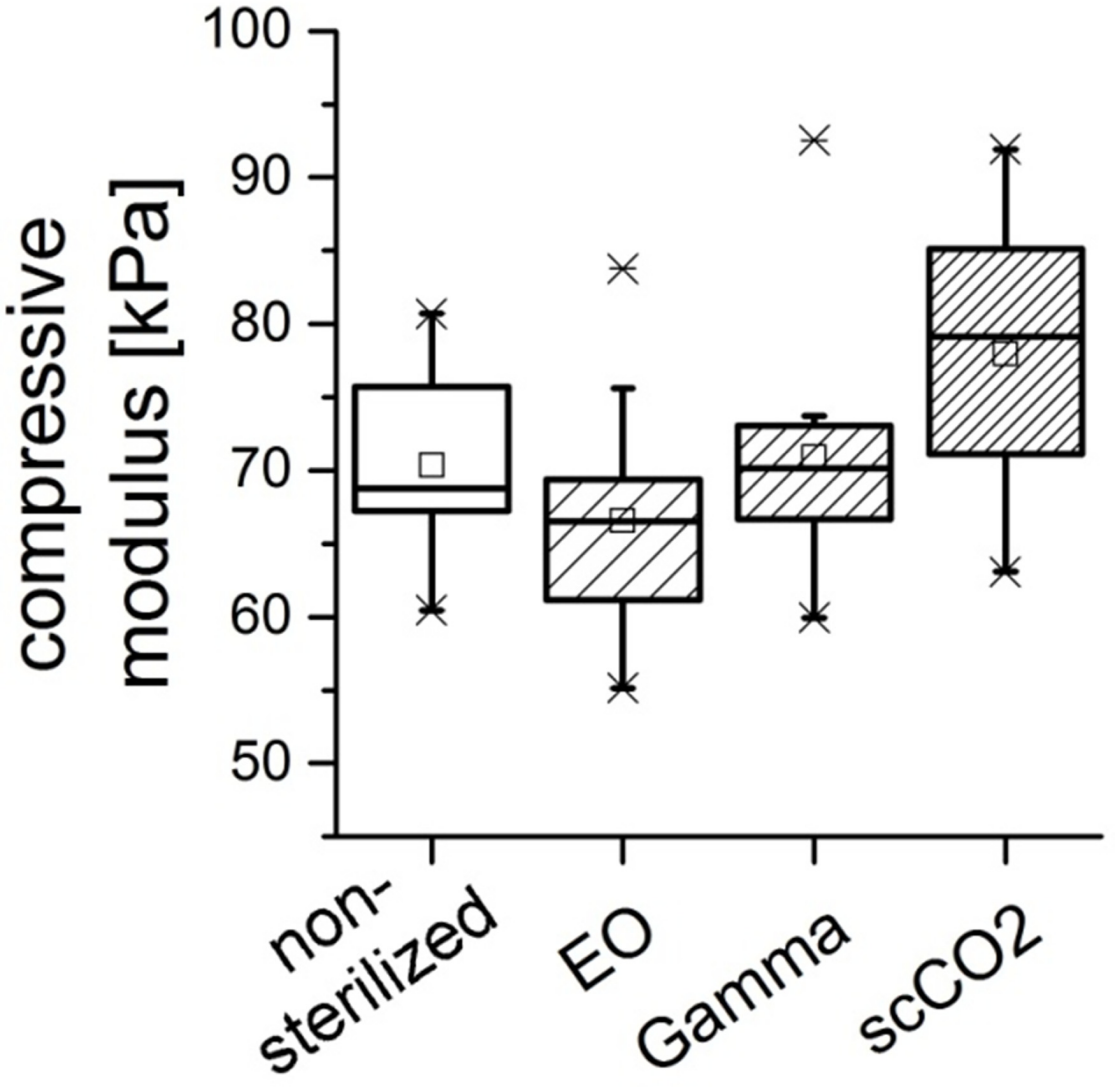
Box plot visualizing the compressive moduli of porous scaffolds from mineralized collagen after treatment with different sterilization procedures. Each box shows the 25th to 75th percentile of the measured modulus. Squares (□) represent mean values (n = 6), horizontal bars inside the box show the median value, while upper and lower bars indicate the upper and lower values within 1.5 times the inter-quartile range from the upper and lower quartile (adjacent values are labelled as ×).

Interestingly, gamma irradiation did not compromise the compressive modulus of fibrillized, mineralized and chemically cross-linked bovine collagen (mineralized collagen scaffolds). In contrast, EO sterilization decreased and scCO_2_ treatment even increased the compressive modulus, which was statistically significant in the presented experiments (p<0.01). (Fig 7)

#### Biphasic collagen scaffolds (Matricart)

**Fig 8:**
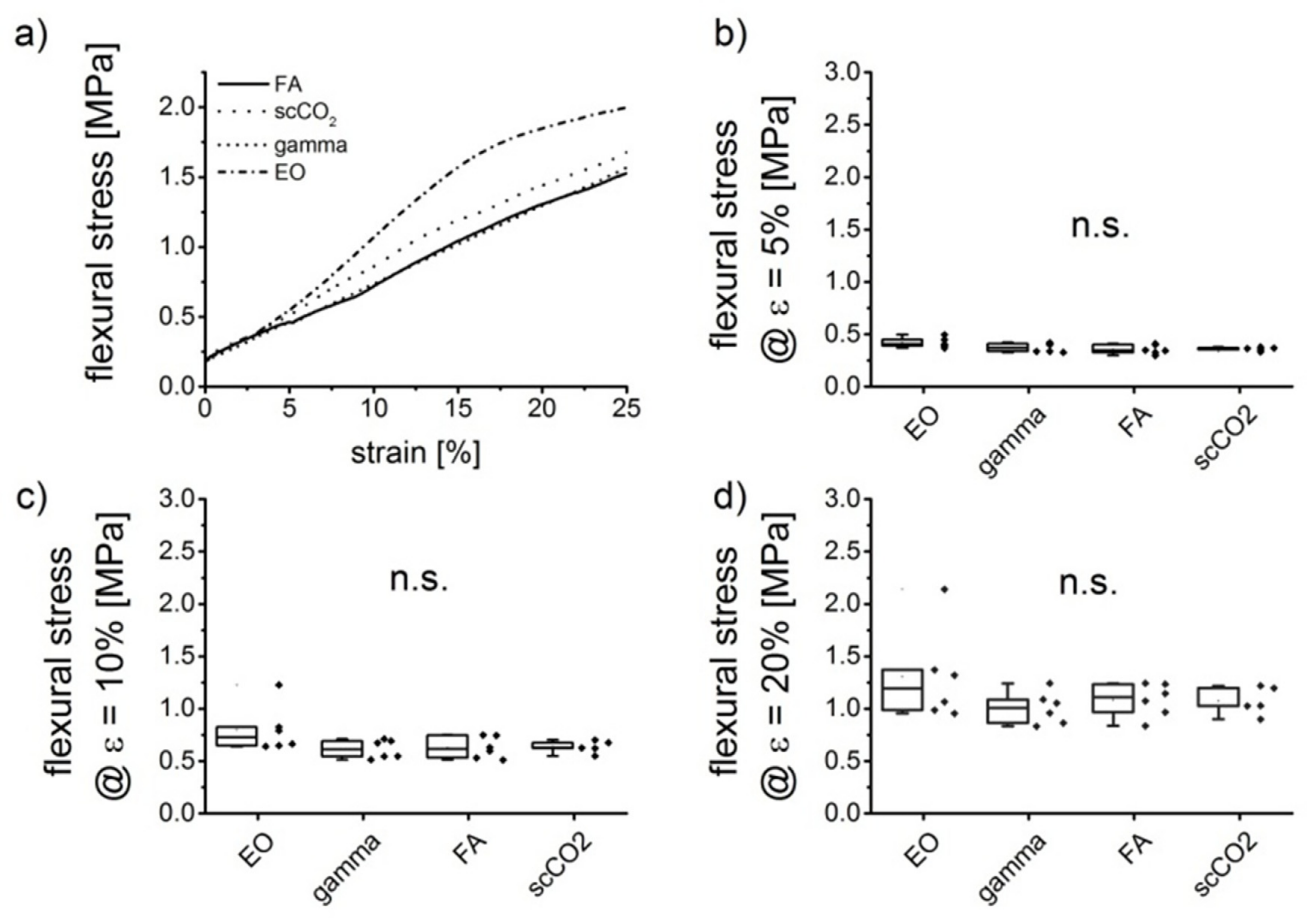
Results of bending strength measurement on differently sterilized samples: representative stress-strain curves (a) and box plots visualizing the bending stress values at 5, 10 and 20% deformation (b-d). Each box shows the 25^th^ to 75^th^ percentile of the measured bending stress. Squares (□) represent mean values (n = 6), horizontal bars inside the box show the median value, while upper and lower bars indicate the upper and lower values within 1.5 times the inter-quartile range from the upper and lower quartile (adjacent values are labelled as ×).

We used EO, gamma irradiation, formaldehyde/steam and scCO_2_ to treat patches made from a micro-porous collagen matrix and a collagen membrane (Matricart) for the preparation of autologous cartilage implants. After sterilization the patches were cut into strips of 3 mm x 1,8 mm x 20 mm and analyzed for their flexural properties (Fig 8). Although a slight increase of flexural stress could be found in case of EO treated samples, no significant influence of the sterilization method on the flexural properties could be found.

### Cytocompatibility of scCO_2_ treated biomaterials

#### Alginate

**Fig 9:**
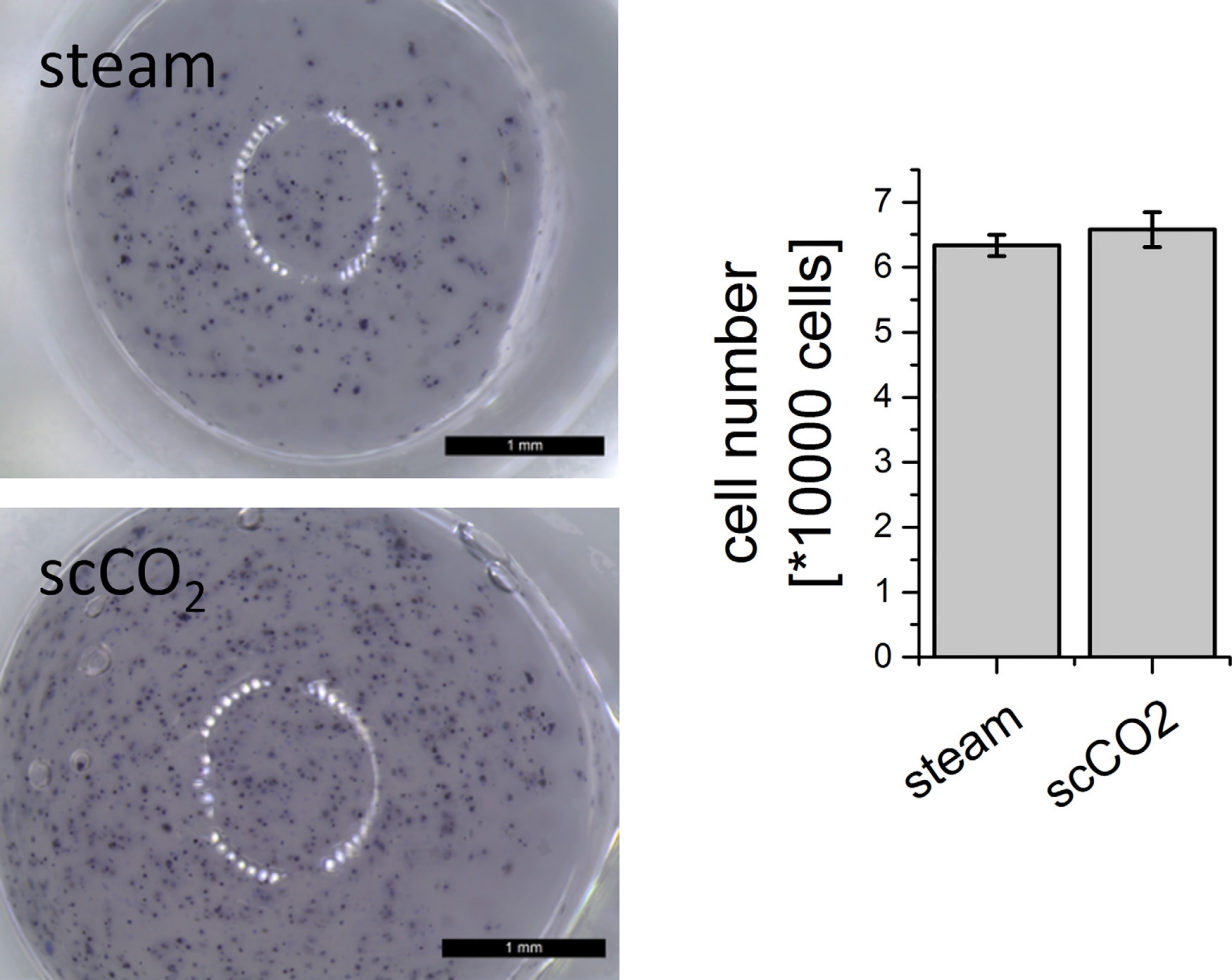
Alginate beads prepared from steam-sterilized and scCO_2_ sterilized powders with embedded hMSC. Left: MTT staining indicates viable cells. Scale bar = 1 mm. Right: Cell numbers calculated from LDH activity after lysis of the embedded cells (n = 4, error bars show standard deviation from the mean).

Alginate beads with embedded hMSC were fabricated from steam-sterilized as well as scCO_2_ treated alginate. After one day of cultivation vital cells were detected visually by MTT staining in both groups (Fig 9). Furthermore, the number of vital cells was evaluated by measurement of LDH activity after lysis of the cells. No significant differences were detected for steam-sterilized and scCO_2_ treated alginate (Fig 9).

#### Mineralized collagen

Porous scaffolds from mineralized collagen were sterilized by EO, gamma irradiation and scCO_2_. Proliferation and osteogenic differentiation of hMSC were analyzed over a period of 4 weeks.

**Fig 10:**
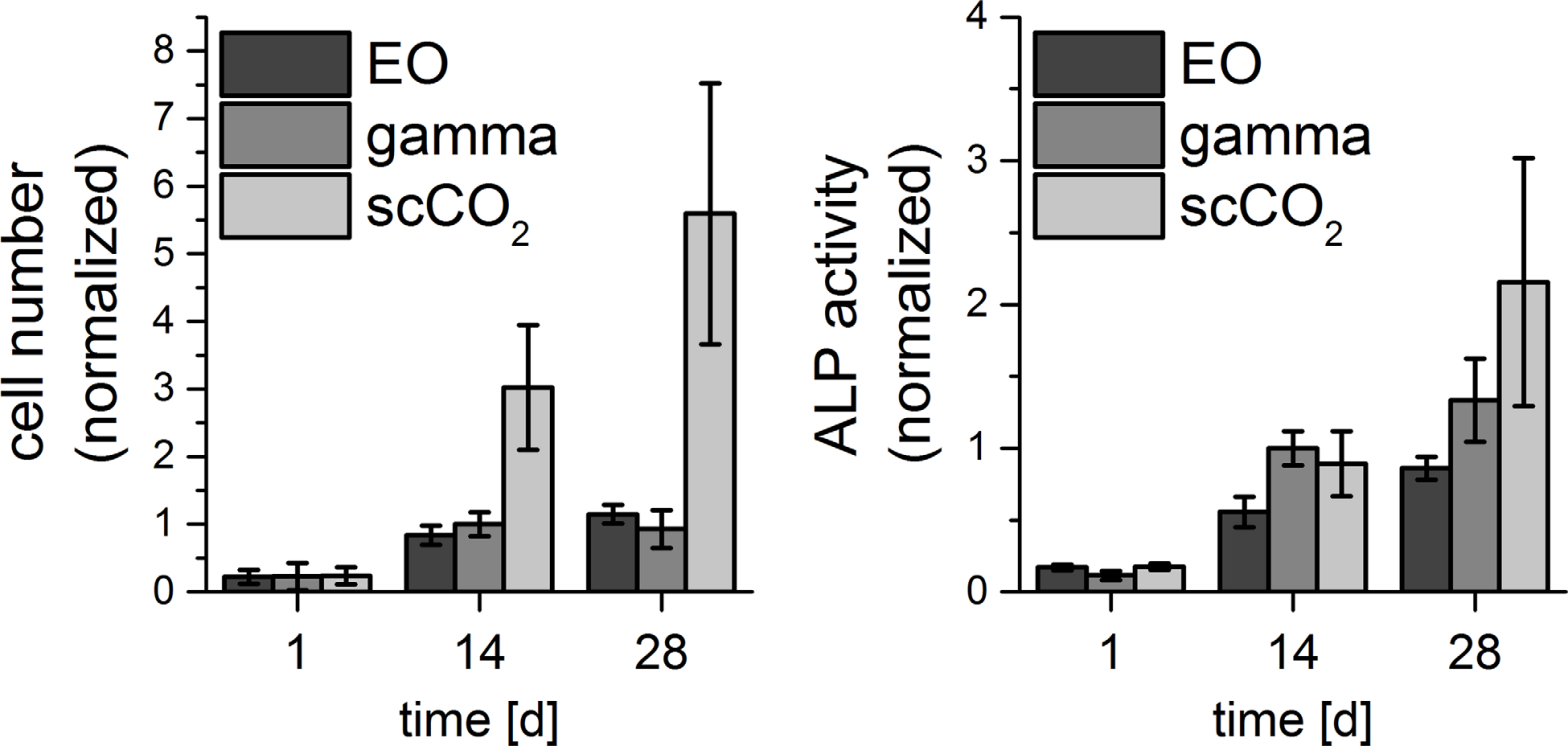
Cell numbers calculated from DNA content and ALP activity of hMSC which were cultivated for up to 4 weeks in porous scaffolds from mineralized collagen under osteogenic stimulation. Scaffolds were sterilized by ethylene oxide exposure, gamma irradiation or scCO_2_. Data of experiments with hMSC of two different donors were combined (n = 6, each donor n = 3). Because of the different proliferation of the cells, data were normalized to d14 values of gamma irradiated samples, which were set to 1. Error bars show standard deviation from the mean (n = 6).

During the first two weeks cell number (calculated from DNA content) increased in all groups. Interestingly, the cell numbers on scCO_2_ treated scaffold were significantly (p<0.001) higher compared to cell numbers on EO sterilized and gamma irradiated scaffolds both after 14 and 28 days of cultivation. Osteogenic differentiation of the cells was evaluated by the determination of ALP activity. ALP activity per scaffold after 28 days of cultivation was significantly higher on scCO_2_ treated scaffolds compared to gamma irradiated (p<0.05) and EO sterilized scaffolds (p<0.01) (Fig 10).

## Discussion

The antimicrobial activity of scCO_2_ has already been described decades ago and was mainly applied for the sterilization of food. The exact mechanism of microbial inactivation by scCO_2_ is not yet elucidated. However, it is hypothesized that special properties of scCO_2_ like low viscosity and high permeability as well as its amphiphilic behavior are responsible for the extraction of cell wall lipids and intracellular compounds, cytoplasmatic pH decrease and inactivation of key metabolic enzymes [6, 8]. During the last decade there have been increasing efforts to utilize scCO_2_ treatment for the sterilization of biomedical devices. In the present study the inactivation of test microorganisms in hydrogel PCD sealed in tyvek-sterile barrier pouches was analyzed. Furthermore, the impact of scCO_2_ sterilization on mechanical properties of different biopolymers and the cytocompatibility of scCO_2_ treated biomaterials were evaluated. It has been demonstrated by others that scCO_2_ alone is not sufficient to inactivate bacterial endospores at ambient temperature and that low molecular volatile additives are indispensable to achieve sufficient inactivation (see introduction). Furthermore, the addition of water that is at least soluble in fractions [30, 31] in scCO_2_ promotes high inactivation rates [5]. It is assumed that water increases the permeability of the cell wall facilitating the diffusion of scCO_2_ [5]. Higher antimicrobial activity of scCO_2_ in the presence of water has been reported by different groups [5, 13]. In the present study, a mixture of acetic anhydride and hydrogen peroxide in water was used for the first time to enhance the microbial inactivation by scCO_2_. We detected a considerably increased reduction of microbial activity compared to scCO_2_ treatment with only hydrogen peroxide in water as additive (data not shown). In the applied additive combination, the three components will react further to yield a mixture of acetic acid, hydrogen peroxide and peracetic acid (PAA) which is known as an effective antimicrobial agent. White and co-workers [7] demonstrated that in such a mixture PAA is the driving force for inactivation of *B*. *stearothermophilus* endospores, followed by hydrogen peroxide, while acetic acid was not very effective. The same group furthermore revealed that PAA in combination with scCO_2_ was 100-fold more effective compared to PAA alone and 5-fold more effective compared to scCO_2_ with hydrogen peroxide. Most investigations on microbial inactivation are performed without embedding the microorganisms prior to sterilization. However, for the sterilization of bulky biomaterials the inactivation in the entire construct is crucial and should be demonstrated when evaluating the potential of a new sterilization method. In the present study, microorganisms were embedded in alginate spheres, which were further embedded in agarose cylinders. Jimenez and co-workers [32] described the inactivation of gram positive and negative bacteria inoculated in a model hydrogel by scCO_2_ treatment. However, no bacterial spores were included into the study. Successful scCO_2_ induced inactivation of *B*. *subtilis* spores inoculated in PEG hydrogels by treatment with relatively harsh conditions (70°C, 4–6 h) was reported by Kaajanagi and co-workers [17]. Interestingly, the water content inside the hydrogels was sufficient to enhance the microbial inactivation and no further additives were necessary. In our study we demonstrated high inactivation rates for bacterial endospores inoculated in hydrogel composites. We conclude that our sterilization method is powerful to inactivate bacterial endospores also inside porous bulky biomaterials. Terminal sterilization of biomedical devices usually requires packaging in a sterile barrier system. It has been shown before, that tyvek, a membrane of flash spun high density polyethylene fibers, which is frequently used for packaging of gamma-, EO- and steam-sterilized devices can be penetrated by scCO_2_ [7, 18, 19]. Therefore, all sterilizations in this study were performed with tyvek-sealed test materials. Furthermore, the conditions which were demonstrated to inactivate bacterial endospores in hydrogel PCD sealed into tyvek pouches (45 min/8.5 MPa/38°C in the presence of 0.25% water/0.15% H_2_O_2_/0.5% acetic anhydride) were considered sufficient for sterilization and were applied for the following tests regarding the impact of scCO_2_ treatment on the mechanical properties and biocompatibility of biomaterials.

To the best of our knowledge, the present study is the first to analyze the mechanical properties of alginate hydrogels after sterilization with scCO_2_ compared to classical sterilization methods. Polysaccharide-based hydrogels are known to be very sensitive to sterilization. Especially gamma irradiation leads to a reduction of molecular weight through chain breaks which is accompanied by a loss of viscosity [33–35]. Recently, gamma irradiation of alginate powder was demonstrated to act more destructive compared to steam sterilization [36, 37]. After gamma sterilization at 20 kGy molecular weight was reduced to 27% while autoclaving for 25 min reduced the molecular weight only to 76% [36]. Furthermore, the same group revealed considerably higher degradation for alginate/hydroxyapatite composites from irradiated alginate compared to those from autoclaved alginate. Accordingly, Hu and co-workers [37] demonstrated a loss of intrinsic viscosity after sterilization of alginate powders with gamma irradiation (47.2% viscosity compared to untreated alginate) and steam sterilization (75.5% compared to untreated alginate). Nevertheless, despite less severely, also steam sterilization of alginate provokes significant changes in the mechanical properties of hydrogels. In that respect, a remarkably reduced elastic modulus of alginate hydrogels [38] and reduced water retention within alginate hydrogels [39] were reported after steam sterilization. In the present study the effect of scCO_2_ sterilization on the mechanical properties of alginate hydrogels was examined. In accordance with the already cited data hydrogels from gamma irradiated alginate showed the highest decrease of compressive strength and modulus, while mechanical parameters for hydrogels of steam sterilized alginate were significantly higher. The lowest decrease of compressive strength and modulus compared to non-treated samples was found for hydrogels prepared from scCO_2_ sterilized alginate. These findings indicate that scCO_2_ treatment might be more gentle compared to steam and irradiation. The advantage over steam sterilization is the reduced temperature, since at moderate temperature of < 40°C degradation mechanisms like acid hydrolysis and the release of free radicals should be considerably reduced [36]. In comparison to gamma irradiation scCO_2_ treatment should not cleave glycosidic bonds within the polysaccharide chain. Valuable results were furthermore obtained by scCO_2_ treatment of methylcellulose (MC). In our study steam autoclaving of methylcellulose powder led to severe agglutinations and therefore, no further experiments were performed with methylcellulose treated in this way. Similarly, inhomogeneous solutions and white precipitates were observed after autoclaving of hydroxypropylmethylcellulose solutions [40]. In our study gamma irradiation of MC caused a significant loss in viscosity of MC/alginate pastes, while the viscosity of methylcellulose/alginate pastes from scCO_2_ treated MC was comparable to the viscosity of non-treated samples. Accordingly, Matthews and co-workers [41] reported drastically reduced viscosity of alginate/MC blends which were irradiated in the dry state and then rehydrated. Interestingly, the reduction of viscosity increased with increasing MC content of the blend indicating a higher sensitivity of MC compared to alginate when subjected to gamma irradiation [41].

Besides polysaccharide-based biomaterials also the sterilization of other biopolymers requires gentle procedures to conserve the structure and mechanical properties of those materials. Collagen, a ubiquitous extracellular matrix protein is frequently used as biomaterial for biomedical applications, like wound healing and tissue repair. Autoclaving is no option for the sterilization of collagen-based biomaterials since collagen is easily denaturized by elevated temperatures. Sterilization using gamma irradiation and EO are superior to steam sterilization, although there are still changes in physical properties caused by non-thermal sterilization techniques [4]. In the present study, the influence of scCO_2_ sterilization on the mechanical parameters of two different collagen-based materials was analyzed in comparison to other sterilization methods. Porous scaffolds from mineralized collagen showed an increased compressive modulus after scCO_2_ treatment, while EO sterilization decreased it and gamma irradiation did not change the compressive modulus. It has been demonstrated earlier, that EO may react with the amino acids lysine and hydroxy-lysine leading to a decreased stability of the collagen helix [42]. The absent impairment of the mechanical strength by gamma irradiation may be attributed to the relatively low irradiation dose (25–30 kGy) in our study. Moreover, collagen was treated in this study after mineralization and chemical cross-linking, which both stabilizes the scaffold. The increase of compressive strength due to scCO2 treatment was surprising. Possibly the grain size of nanocrystalline hydroxylapatit was changed under the elevated pressure. Further transmission electron microscopy investigations are needed to clarify this assumption. Sterilization of biphasic collagen patches, comprising of a collagen membrane and a microporous collagen matrix did not reveal significant effects on mechanical parameters. Flexural strength of the constructs was not significantly different after sterilization with ethylene oxide, gamma irradiation, formaldehyde/steam and scCO_2_. Our results on the influence of sterilization on mechanical properties of biopolymers indicate that the highest benefit of scCO_2_ sterilization is obtained for polysaccharide based materials, while collagen-based materials may also be sterilized with gamma irradiation without significant loss of stiffness and flexural strength.

A very important concern is the impact of sterilization on cytocompatibility of the treated materials. This is especially important for chemical sterilization methods like EO and formaldehyde/steam since remaining residues could compromise the biocompatibility of the sterilized materials. However, also the application of organic additives in the scCO_2_ sterilization process needs to be examined for possible cytotoxic effects. Chang and co-workers [43] analyzed the cytotoxicity of bone allograft powder after sterilization with scCO_2_. No cytotoxic effect was detected on L929 fibroblast cells; however, this study was performed without organic additives. Studies on the impact of sterilization on cellular behavior are rare, since most studies analyze the influence of sterilization on structural and chemical properties of the treated materials. A recently published study investigated the influence of gamma irradiation, ethylene oxide and disinfection with PAA on initial cell adhesion and proliferation on decellularized collagen-based scaffold [44]. Interestingly, both sterilization methods provoke structural changes in this special scaffold compromising cellular adhesion and proliferation, while PAA disinfected scaffolds showed the highest cell numbers both after 1 and 4 days of *in vitro* cultivation. A decreased proliferation of endothelial cells in the presence of steam sterilized alginate compared to gamma irradiated alginate was reported recently [36] however, no explanation was provided for this phenomenon. Wehmeyer and co-workers [45] analyzed the proliferation of human MSC on scCO_2_ treated amniotic membrane tissue and did not find any cytotoxic effects when using PAA as additive for scCO_2_ sterilization. We conclude from these and our results, that scCO_2_ sterilization under addition of low levels of PAA and other low molecular organic compounds does not induce cytotoxic effects of tissue samples, collagen- and polysaccharide-based materials. The present study is limited to *in vitro* biocompatibility investigations. It is of great importance to examine the effect of scCO_2_ sterilized materials also *in vivo*. Russell and co-workers analyzed the impact of gamma sterilized and scCO_2_ treated (PAA and H_2_O_2_ as additives) cortical bone allografts on early inflammation and early bone formation [23] and even found an increased new bone formation around scCO_2_ treated allografts compared to gamma irradiated ones. This effect can be contributed to the preserved bioactivity of tissue-specific growth factors and is not expected to be found with scCO_2_ sterilized synthetic biomaterials. Nevertheless, the high biocompatibility of scCO_2_ treated scaffolds in vitro, which was demonstrated in this study, is no guarantee for high biocompatibility in vivo. Furthermore, the level of residual additives in the porous structures should be checked before starting in *vivo* investigations.

## Conclusion

Sterilization with scCO_2_ under addition of 0.25% water, 0.15% hydrogen peroxide and 0.5% acetic anhydride successfully inactivated a broad panel of microorganisms including bacterial endospores, even when embedded in hydrogel PCD and sealed in tyvek pouches. Mechanical properties of polysaccharide- and collagen-based biomaterials were less compromised by scCO_2_ compared to classical sterilization methods. Especially in the case of methylcellulose rheological parameters were not affected by scCO_2_ sterilization while viscosity of gamma irradiated samples was dramatically decreased. Since no cytotoxic effects of the additives were detected in vitro, scCO_2_ sterilization with the suggested procedure is a promising alternative to already established sterilization methods. Further investigations will include other sensitive biomaterials, like chitosan and silk fibroin, the treatment of scaffolds loaded with functional proteins which are sensitive to sterilization, and biocompatibility tests *in vivo*.
